# Emotions Meet Reflexivity in Workplace Training: A Person-Centered Approach to Understanding Transfer of Learning

**DOI:** 10.3390/bs16071048

**Published:** 2026-06-23

**Authors:** Eleonora Cova, Maria Luisa Farnese

**Affiliations:** 1 Department of Development and Social Psychology, Sapienza University of Rome, 00185 Rome, Italy; eleonora.cova@uniroma1.it; 2 Department of Psychology, Sapienza University of Rome, Via dei Marsi 78, 00185 Rome, Italy

**Keywords:** emotion, reflexivity, transfer of learning, training

## Abstract

This study examines how emotional and reflexive processes jointly relate to transfer of learning in workplace training contexts. Drawing on organizational learning theory, it introduces Reflexivity on Emotions (RoE) as a metacognitive capability through which individuals become aware of, critically examine, and respond to their emotional experiences. Integrating RoE, reflexivity on practice, positive affect, and negative affect within a person-centered framework, the study applies Latent Profile Analysis (LPA) to data collected from 609 correctional officer cadets enrolled in a six-month training program. The analysis identified four emotional–reflexive profiles (*Generative–Reflexive*, *Balanced–Reflexive*, *Detached–Unreflexive*, and *Inhibited–Unreflexive*), which showed different levels of transfer of learning. Notably, the Generative–Reflexive profile, characterized by elevated negative affect alongside strong reflexive resources, was associated with the highest levels of transfer, suggesting that negative emotions are not uniformly associated with poorer learning outcomes. More broadly, the findings indicate that transfer of learning is better understood through emotional–reflexive configurations rather than through isolated factors. The study contributes to organizational learning research by extending reflexivity into the emotional domain and by demonstrating the value of person-centered approaches for understanding individual differences in workplace learning. Practical implications for training design and the development of emotionally reflective learning environments are discussed.

## 1. Introduction

Training has a significant impact on individuals’ lives and careers, whether by enabling them to learn new competencies, enhancing self-awareness and life perspectives, or fostering personal change (Merriam & Clark, 1993). Given the transformative nature of learning processes, emotions represent a fundamental dimension of training experiences, influencing both participants’ engagement and the effectiveness of transfer of learning (e.g., Benozzo & Colley, 2012; Rowe & Fitness, 2018). Accordingly, emotions are not merely affective responses but constitute interpretive processes that shape professional meaning-making (Ashkanasy & Dorris, 2017) and learning (Vince, 2022). Indeed, effective training in complex workplaces requires more than content delivery: it relies on learners’ ability to make sense of their experiences, engage emotionally (Bulut & Culha, 2010), and consistently adapt their practices.

In light of this, the metacognitive capability to reflect on one’s own emotions, namely Reflexivity on Emotion (RoE), may support individuals in increasing awareness and understanding of their emotional experiences during complex emotional events (Cova & Farnese, 2025) occurring within training processes, thereby facilitating effective transfer of learning. Specifically, RoE may serve as a conscious and deliberate tool to regulate emotions (Travers et al., 2020; Mendonça & Sàágua, 2019), transforming them into meaningful insights that guide current responses and inform future actions (Hibbert et al., 2022). In turn, emotional experiences, when properly reflected upon, can be leveraged as resources for learning.

While prior research on the learning–reflexivity relationship has primarily focused on reflexivity related to cognitive processes (e.g., decision-making) and work practices (e.g., questioning goals, selecting methods, or managing tasks and procedures) (see the meta-analysis by Leblanc et al., 2024), the heterogeneity of emotional–reflexive experiences during training has been largely overlooked. Moreover, these studies have largely adopted a variable-centered approach, with only a few exceptions (M.-C. Wang et al., 2017; Lee & Chei, 2020), thereby limiting the possibility of capturing the more nuanced and configurational aspects of these processes. To address these gaps, the present study examines how distinct emotional–reflexive profiles are associated with varying levels of transfer of learning.

By investigating reflexivity across both the practice and emotional domains, this study makes two main contributions. First, it extends the reflexivity literature by demonstrating the relevance of reflexivity not only for work practices but also for emotional experiences in learning processes, responding to recent calls for a more nuanced understanding of emotional–reflexive configurations (Rentzios et al., 2025; Xu et al., 2024). Second, it contributes to the organizational learning literature by improving understanding of reflexivity as a key mechanism supporting the transfer of learning, especially in emotionally demanding learning contexts.

## 2. Theoretical Framework

### 2.1. The Role of Reflexivity in Learning

Reflexivity has its roots within organizational learning theory, which conceptualizes self-reflexivity as a capability that, through deliberate engagement in inquiry, leads to the development of new knowledge (Cunliffe, 2016; Mezirow, 2015; Schön, 1983; McGarry, 2019). It involves suspending habitual patterns of action to create purposeful “pauses” with transformative potential (Fels, 2015; Noble et al., 2025), a performative process that enables movement, repositioning, and change (Aoki, 1996; Beers & van Mierlo, 2017). Beyond merely reflection about a problem, reflexivity is a metacognitive capability that enables individuals to critically examine and question their own beliefs and established routines. Its intentional and transformative nature distinguishes it as a process that is “more than reflection” (Holmes, 2010, p. 140).

Furthermore, reflexivity does not occur in a vacuum. As Archer (2003) argues, it originates in an internal conversation that serves as a bridge between structure (the social context that enables and constrains social actions) and agency (individuals’ capability for proactive action). Therefore, reflexivity is both shaped by the social context in which individuals are embedded and actively contributes to its reframing, encompassing individuals’ ongoing ability to continuously shape and reorganize their behaviors based on their evolving understanding of the social world.

Drawing on this foundational literature, organizational research has extended the concept of reflexivity to collective dynamics, focusing on team reflexivity and highlighting its value across several aspects of working life, as evidenced by the meta-analysis conducted by Leblanc et al. (2024). Specifically, it hinders the risk of groupthink, enhances team effectiveness (Brav et al., 2009; Farnese & Livi, 2016; Jonasson & Lauring, 2026), fosters creativity (West & Sacramento, 2006; Rong et al., 2019), and innovation (Widmer et al., 2009; Farnese & Livi, 2016; Chen et al., 2019).

Importantly, reflexivity also plays a crucial role in learning (Harvey et al., 2023; De Dreu, 2007; Matsuo, 2018; West & Sacramento, 2006; Baerheim & Ness, 2021). While organizational scholars unanimously acknowledge the link between learning and reflexivity, various theoretical perspectives conceptualize this relationship in distinct ways. Four main perspectives can be identified: (a) reflexivity and learning as components of a unified process (Edmondson, 1999); (b) learning as a constituent process of reflexivity (West, 2000; Widmer et al., 2009); (c) learning as a process that, under certain conditions, fosters reflexivity (Beers & van Mierlo, 2017); and (d) reflexivity as a precursor to learning (e.g., Gherardi et al., 2018; Matsuo, 2018; Wals et al., 2004). From the standpoint of organizational learning, this latter perspective conceptualizes reflexivity as a mechanism that facilitates learning. Empirical evidence supports this view, showing that intentional reflexive practices at both individual and team levels tend to precede and enable learning processes.

Notably, organizational learning has long been conceptualized as a process through which individuals or groups “acquire, interpret, reorganize, change, or assimilate clusters of related information, skills, and emotions” (Marsick & Watkins, 1990, p. 4), thus explicitly acknowledging the relevance of emotional experiences within learning dynamics. However, despite this broad conceptualization, research on the reflexivity–learning relationship has predominantly focused on practices, cognitions, and behaviors, largely overlooking the emotional domain. This imbalance points to the need for a more integrative understanding of reflexivity that also encompasses how individuals engage with and make sense of their emotional experiences during learning processes.

Although an emerging body of literature points to the relevance of reflexivity on emotions (RoE) within this dynamic (Quinn & Bunderson, 2016; Grisoni, 2017), empirical evidence remains limited and fragmented, primarily adopting qualitative approaches. Within this emerging line of research, RoE can be defined as a metacognitive capability through which individuals reflect upon, question, and critically examine their emotional experiences (Holmes, 2015; Burkitt, 2012; Rosenberg, 1990), thereby fostering greater self-awareness and supporting learning processes (Cova & Farnese, 2025). In particular, Quinn and Bunderson (2016) showed that reflexivity enables participants to explore both convergence and divergence in perspectives, playing a crucial role in making sense of both informational and emotional cues during team interactions. This process not only supports deeper learning but also strengthens interpersonal relationships, as participants engage in attentive efforts to understand both their own and others’ emotional experiences. Similarly, Grisoni (2017) showed that engaging in reflexivity on both experiences and emotions allowed employees to mobilize their imagination and develop a more nuanced understanding of others’ perspectives and emotional responses, thereby facilitating deeper learning processes and supporting both personal and professional development.

### 2.2. The Role of Emotion in Learning

Scholars widely agree that positive emotions—such as happiness, pride, and hope—constitute a core motivational foundation for learning, as they foster engagement, persistence, and cognitive openness (Pekrun et al., 2002; Li et al., 2020). Accordingly, higher levels of positive affect are generally associated with enhanced learning outcomes. Conversely, negative emotions are often associated with psychological strain and may interfere with learning processes. According to coping theory (Lazarus & Folkman, 1984), when individuals experience intense negative affect, their regulatory resources tend to be directed toward emotion-focused coping strategies rather than problem-focused ones. Because these strategies primarily aim to reduce emotional distress rather than address the underlying issue, they may constrain opportunities for reflection, adaptation, and learning.

Empirical evidence supports this perspective. For instance, Brown et al. (2005) found that emotion-focused strategies, such as venting negative emotions to others, were negatively related to performance, whereas problem-focused or task-oriented strategies led to more positive outcomes. Similarly, Schwarz and Bless (1991) argued that intense negative affect narrows attention, leading individuals to select fewer demanding tasks, thereby reducing learning opportunities.

However, the relationship between affect and learning is more complex than a simple linear one (Pekrun & Stephens, 2010). Negative emotions may actually serve an adaptive function by improving performance (Rowe & Fitness, 2018). Furthermore, Efklides and Petkaki (2005) reported that negative emotions can prompt students to examine learning-related problems more carefully, whereas positive emotions are associated with holistic thinking. This suggests that negative emotions should not be viewed solely as states to be avoided, but rather as experiences that learners may need to face and regulate.

Understanding learning processes, therefore, requires considering how emotions shape them (Pekrun et al., 2002). Specifically, although prior literature has traditionally approached this issue through a dichotomy between positive and negative emotions, the picture is more nuanced. In line with Lee and Chei (2020), positive and negative emotions should not be viewed as opposite ends of a single continuum, but rather as relatively independent dimensions that may coexist within the same emotional event. Their interplay gives rise to distinct emotional profiles, reflecting a multi-emotion perspective in which individuals simultaneously experience a range of positive and negative affective states (e.g., Linnenbrink & Pintrich, 2002; Pekrun et al., 2002; Song & Choi, 2011).

### 2.3. Emotional–Reflexive Profiles in Learning

In an effort to better account for the variability in how emotional experiences relate to learning outcomes, recent research has increasingly shifted toward examining configurational patterns of emotions and regulatory processes, rather than treating these elements in isolation (e.g., Rentzios et al., 2025; Xu et al., 2024). This perspective recognizes that individuals differ not only in the valence and intensity of the emotions they experience, but also in how they process and integrate these experiences. While treating learners as a homogeneous group may obscure meaningful differences and limit the development of tailored learning interventions, studying distinct profiles allows for a more granular understanding of how the interplay of different emotional states and regulatory mechanisms may lead to different learning outcomes across individuals.

In this vein, recent research has increasingly adopted person-centered approaches to identify subgroups of trainees with similar profiles, showing that multiple factors can jointly contribute to learning and performance outcomes (Rentzios et al., 2025; Lee & Chei, 2020). Accordingly, a more comprehensive understanding requires moving beyond the isolated study of emotions to explore how they interact with reflexive processes, as this interplay may ultimately determine whether and how learning is effectively translated into practice. As noted by Pekrun (2024), capturing the complexity of learning requires the meaningful integration of multiple constructs. Such an integrative perspective is particularly valuable for explaining why certain groups of learners do not align with dominant theoretical expectations, typically associated with positive affect, yet still achieve satisfactory outcomes.

Empirical evidence supports this view. For instance, Heikkilä et al. (2012) identified groups of non-academic, self-directed, and helpless learners who, despite not exhibiting prototypically successful profiles, were able to progress in their studies. Similarly, Jarrell et al. (2017) found that students reporting low-valence emotions could still achieve good academic outcomes, while Postareff et al. (2017) identified learners characterized by negative emotions who nonetheless adopted deep approaches to learning and made significant progress. Other studies showed that anxiety does not necessarily hinder performance, and may, under certain conditions, support it (Z. Wang et al., 2018), and that students experiencing high levels of negative affect can still demonstrate strong academic performance (Robinson et al., 2017).

Taken together, these findings highlight the complexity of emotional profiles, showing that negative emotions can coexist with adaptive learning strategies and satisfactory outcomes. This underscores the importance of examining configurations of cognitive and emotional factors when investigating the quality of learning (Heikkilä et al., 2012).

In an attempt to account for the apparent dissonance between emotional experiences and learning outcomes, recent studies have begun incorporating regulatory and reflective processes into their analysis of these configurations. In particular, constructs such as reflective thinking (Rentzios et al., 2025) and reflective learning (Xu et al., 2024) have been proposed as potential mechanisms through which individuals reinterpret and regulate their experiences, thereby sustaining learning performance even under less favorable emotional conditions.

Within this perspective, RoE represents a particularly relevant mechanism. By engaging in the reflective regulation of their emotional experiences, individuals may transform the potential costs of negative affect into a resource for learning, balancing hedonic concerns with goal-directed regulation. However, a more integrative perspective suggests that individuals are not uniform in their reflexive capacities: some may excel at critically examining their practices, others at navigating emotional experiences, and others at combining both domains (Schel & Drechsel, 2025). From this standpoint, adopting a person-centered approach becomes essential, as it enables the identification of distinct reflexive configurations and provides a deeper understanding of how RoE and reflexivity on practice jointly shape learning processes.

### 2.4. The Present Study

To summarize, this study distinguishes RoE from reflexivity on practice. The latter refers to the metacognitive capacity to critically examine and question one’s own practices, actions, and behaviors to revise established routines. In contrast, RoE directs toward affective experiences, enabling individuals to make sense of their emotional responses and learn from them. In this regard, RoE can be conceptualized as a deliberate, learning-oriented form of emotional regulation, through which emotions are explored as sources of insight and development rather than managed solely for hedonic purposes. Importantly, although conceptually distinct, these two forms of reflexivity are likely to operate in conjunction with individuals’ emotional experiences, giving rise to distinct patterns of adaptation and learning.

Thus, building on emerging evidence suggesting that learning is not shaped by emotions or reflexivity alone, but rather by their specific configurations, this study aims to provide a more nuanced representation of individual differences in learning. Specifically, we adopt a person-centered approach to identify emotional–reflexive profiles that integrate both forms of reflexivity (i.e., on practice and on emotions) and affective experiences (i.e., with positive and negative valence).

Based on prior literature, it is reasonable to expect that different emotional–reflexive profiles may emerge from the joint configuration of reflexivity on practice, reflexivity on emotions, and affective experiences, differentially shaping the extent to which learning is effectively translated into practice. First, consistent with traditional learning perspectives, an *adaptive profile* can be characterized by adequate levels of reflective engagement both with practice and emotions, alongside predominantly positive affect. Individuals in this profile are likely to engage in proactive reflection when needed, enabling them to regulate and counterbalance negative experiences while sustaining a positive motivational state, thereby supporting effective learning outcomes. Second, an *impaired profile* may be identified, characterized by low levels of both forms of reflexivity, coupled with high negative affect. In line with classical views on affect and learning, the lack of reflexive capabilities, combined with emotional strain, may constrain individuals’ ability to process experiences constructively, thereby hindering learning outcomes. However, drawing on more recent research highlighting the generative potential of negative emotions—and moving beyond a dichotomous view of such emotions as exclusively detrimental—a third profile may be proposed. This configuration, defined as a *tension-enabled profile*, can be characterized by the experience of negative affect alongside the presence of reflexivity, particularly in its emotion-focused form. In this case, negative emotional experiences may act as informative signals that, when processed reflexively, can support engagement and learning, leading to generative learning outcomes.

Taken together, we advance the following hypotheses:

**H1.**  *When simultaneously considering positive and negative emotions alongside both forms of reflexivity, at least three distinct reflexive–emotional profiles will emerge.*

**H2.**  *These reflexive–emotional profiles will show differential relations with learning outcomes. Specifically, the adaptive profile (characterized by high reflexivity and predominantly positive affect) is expected to report the highest levels of transfer, whereas the impaired profile (characterized by low reflexivity and high negative affect) is expected to report the lowest levels. The tension-enabled profile (characterized by elevated negative affect alongside strong reflexive resources, particularly those oriented toward emotional experiences) is expected to show intermediate, yet still positive, levels of transfer.*

## 3. Materials and Methods

### 3.1. Research Context, Procedure, and Sample

This study was conducted in the Italian Prison System, a complex organization that, since 1975, has expanded its traditional custodial function to include the constitutional goal of promoting inmates’ social reintegration. Within this framework, rehabilitative treatment is implemented through a range of activities aimed at facilitating detainees’ reintegration into society. In this context, correctional officers are responsible not only for maintaining safety and order, but also for contributing to the supervision and rehabilitative processes of detainees. Correctional work is widely recognized as highly stressful (Farnese et al., 2017; Schaufeli & Peeters, 2000), partly due to its inherent role ambiguity: the dual functions of custody and care generate role conflict that increases strain, thereby requiring emotional labor to manage these tensions. This strain is further intensified by frequent exposure to emotionally charged events (Clements & Kinman, 2021), leading officers to engage in emotional dirty work to manage inmates’ emotions, which are socially constructed as “emotional dirt” within custodial settings (Mikkelsen, 2022).

This study involved correctional officers who attended a six-month training program, including lessons at the Prison Police Academy, online instruction, and an eight-week internship in correctional facilities. In July 2023, a ministerial circular informed all training schools involved in the 183rd Training Course about the study and invited them to participate. The circular potentially reached approximately 1700 trainee correctional officers across seven training schools. However, participation was entirely voluntary at the institutional level, with each school commander independently deciding whether to administer the survey. Four of the seven eligible schools chose to participate. Because the survey administration was managed directly by the Penitentiary Administration, the research team did not have access to the number of trainees enrolled in each participating school; therefore, a precise response rate could not be calculated.

During the final week of the training program, after completing the internship period—constituting the primary opportunity for participants to experience and apply the competencies acquired during training—trainees were invited to voluntarily complete an anonymous questionnaire regarding the training they had just completed. This stage of professional socialization was considered particularly relevant for the purposes of the study. As newcomers entering a demanding occupational environment, trainees were likely to be especially attentive to learning processes and to the acquisition of professional competencies. At the same time, their initial exposure to correctional work may have heightened the salience of emotionally challenging experiences, increasing the need to make sense of and manage the negative emotions elicited by the work context. This combination of heightened learning orientation and emotional exposure makes the training period a particularly informative context for examining the interplay between emotions, reflexivity, and transfer of learning. A total of 609 participants completed the survey, and no missing data were present in the final dataset. Participants were mostly men (75.2%), with a mean age of 25.32 years (SD = 2.89).

The study received ethical approval from the Ethics Committee of the authors’ institution and adhered to the APA ethical guidelines and the Declaration of Helsinki.

### 3.2. Measures

*Reflexivity on Emotions* (RoE) was assessed using a 9-item scale developed from an extensive review of existing qualitative studies on reflexivity on emotions (Cova & Farnese, 2025). The instrument captures individuals’ ability to be aware of emotions arising in both routine and emotionally challenging situations, reflect on their emotional responses, and derive insights to inform future behavior. Sample items include “After experiencing an emergency situation, I try to understand the reasons behind my emotional reactions” and “I take time to reflect on the emotional states that the work environment most frequently triggers in me”. Items were rated on a 5-point Likert scale ranging from 1 (Completely false for me) to 5 (Completely true for me). The scale showed excellent internal consistency (Cronbach’s α = 0.91).

*Reflexivity on practices* was measured using a 9-item scale adapted from Schippers et al. (2007), assessing the extent to which individuals critically reflect on, question, evaluate and learn from their work methods, strategies, and practices. Sample items include: “When necessary, I adapt the methods I use based on the situation I was facing” and “I try to learn from past experiences”. Responses were recorded on a 5-point Likert scale ranging from 1 (Strongly disagree) to 5 (Strongly agree). The scale showed good internal consistency (α = 0.80).

*Transfer of learning* was assessed using Tesluk et al.’s (1995) three-item scale, which measures the extent to which knowledge and skills acquired during training are effectively implemented in the workplace, thereby serving as a behavioral indicator of learning effectiveness (Broad & Newstrom, 1992). Respondents indicated their agreement on a 5-point Likert scale from 1 (Strongly disagree) to 5 (Strongly agree). Example items include: “I internalized new professional practices to be used on the job”. The scale showed excellent internal consistency (Cronbach’s α = 0.93).

*Positive Affect and Negative Affect* were each measured using four items from the short form of the Positive Affect–Negative Affect Schedule (PANAS; Thompson, 2007). These scales assess the frequency of emotional experiences at work (“During the last month, I felt […] on the job”). Internal consistency was acceptable, with Cronbach’s alpha values of 0.62 for Positive Affect (i.e., active, proud, determined, and enthusiastic) and 0.84 for Negative Affect (i.e., nervous, angry, worried, and irritable). Responses were recorded on a 5-point Likert scale ranging from 1 (Never) to 5 (Always).

### 3.3. Analysis

Preliminary analyses included descriptive statistics and Pearson correlations among the study variables. To examine the distinctiveness of the two reflexivity constructs, latent correlations with 95% confidence intervals were estimated (Rönkkö & Cho, 2022). To investigate emotional–reflexive profiles, we employed Latent Profile Analysis (LPA), a person-centered approach that complements traditional variable-centered approaches (Marsh et al., 2009). LPA assumes population heterogeneity and identifies subgroups of individuals who share similar configurations across variables. Widely applied in educational, behavioral, and social sciences (Lovegrove & Cornell, 2014; Reichert, 2017), LPA shifts the focus from relationships among variables to patterns within individuals, enabling simultaneous exploration of both commonalities and differences within a sample. Moreover, LPA provides probabilistic classification of individuals into latent profiles, often yielding more objective subgroup delineations than traditional clustering techniques.

Model selection in LPA was guided by multiple statistical criteria. First, the Bayesian Information Criterion (BIC) and sample-size-adjusted BIC (saBIC) were examined, with lower values indicating better model fit. Second, model comparisons relied on the Lo–Mendell–Rubin likelihood ratio test (LMR) and the bootstrapped likelihood ratio test (BLRT), where significant results indicate that a k-class model outperforms a k–1 class solution (Vermunt & Magidson, 2002). Third, entropy values, ranging from 0 to 1, were assessed, with values ≥0.80 indicating good separation between profiles (Muthén, 2004). Finally, class sizes were assessed to ensure that each profile comprised a meaningful proportion of the sample (i.e., >1%; Marsh et al., 2009). All analyses were conducted using the R platform (version 3.1; R Foundation for Statistical Computing, Vienna, Austria).

## 4. Results

### 4.1. Preliminary Analysis

A one-factor CFA of the RoE scale demonstrated good model fit (χ^2^ = 79.801, df = 27, *p* < 0.001; CFI = 0.959; TLI = 0.945; RMSEA = 0.057; SRMR = 0.037), with standardized factor loadings ranging from 0.57 to 0.82, supporting the structural validity of the construct.

To further examine the distinctiveness of RoE from reflexivity on practice, we estimated the latent correlation between the two constructs. As reported in Table 1, RoE was positively associated with reflexivity on practice (r = 0.58, 95% CI [0.53, 0.63]). Although the constructs were moderately related, the estimated correlation remained well below the 0.80 threshold commonly used to indicate a lack of discriminant validity (Rönkkö & Cho, 2022). Taken together, these findings support the conceptualization of RoE and reflexivity on practice as related but empirically distinct dimensions of reflexivity.

Descriptive statistics and correlations among the study variables are reported in Table 2. With regard to the nomological network of the two forms of reflexivity, both RoE and reflexivity on practices were positively associated with transfer of training (respectively, r = 0.238, *p* < 0.001 and r = 0.306, *p* < 0.001). Furthermore, both forms were associated with positive affect (respectively, r = 0.210, *p* < 0.001 and r = 0.212, *p* < 0.001), whereas only RoE showed a marginal association with negative affect (r = 0.091, *p* < 0.05). Finally, transfer of training was positively associated with positive affect (r = 0.304, *p* < 0.001) and negative related to negative affect (r = −0.186, *p* < 0.001). Overall, these findings suggest a limited role of negative affect in both reflexive processes and transfer of learning, while pointing to potential differences in how emotional experiences relate to the two forms of reflexivity.

### 4.2. Emotional–Reflexive Profiles

To capture the joint interplay between emotional experiences and reflexive processes, we adopted a person-centered approach. A Latent Profile Analysis (LPA) was conducted to identify subgroups of individuals characterized by distinct configurations of positive and negative emotion, and the two forms of reflexivity, thereby enabling a more nuanced examination of the study hypotheses.

Given that the AIC, CAIC, BIC, and SABIC indices decreased as additional profiles were added (Table 3), we opted to supplement our decision-making process with the graphical examination of an “elbow plot” to decide the optimal number of profiles to extract.

**Figure 1:**
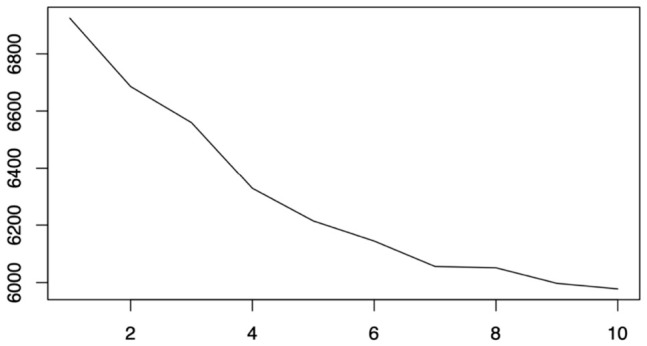
Elbow plot for information criteria. *Note.* The number of profiles extracted in an LPA model is on the X-axis and the fit statistics are on the Y-axis.

In selecting the optimal number of latent profiles, we considered both statistical indices and their graphical representation. The elbow showed evident or slight flattening of the slope around 4 profiles and 7 profiles (Figure 1). In addition, the four-profile model yielded markedly higher entropy (0.89) than alternative solutions, suggesting greater classification accuracy (M.-C. Wang et al., 2017). Additionally, the average latent profile probabilities for the most likely profile were 0.91, 0.88, 0.94, and 0.94, suggesting adequate separation among the four profiles (Spurk et al., 2020). Based on these complementary criteria, the four-profile solution was retained as the most parsimonious and interpretable model, in line with H1. Model fit indices for solutions ranging from one to eight profiles are reported in Table 3.

Accordingly, four emotional–reflexive profiles were identified. The profile labels were selected as heuristic descriptors based on the observed combinations of emotions and reflexivity. They are intended to facilitate interpretation and should not be considered direct representations of latent psychological types or underlying psychological mechanisms. Specifically, three of the profiles align with the theoretically proposed configurations: the *Inhibited–Unreflexive* profile reflects the impaired configuration; the *Generative–Reflexive* profile reflects the tension-enabled configuration; and the *Balanced–Reflexive* profile reflects the adaptive configuration. A fourth profile, labeled *Detached–Unreflexive*, emerged empirically beyond the theoretical configurations initially proposed. Table 4 presents the standardized means of reflexivity and affect variables across the four profiles.

The majority of participants (Cluster 4, 71.9%) were classified within the normative profile, labeled *Balanced–Reflexive*, indicating a general tendency toward mid-range levels across all variables. Adequate levels of reflective engagement with both practice and emotions, alongside moderate levels of both positive and negative affect, suggest that individuals in this profile tend to regulate and respond adaptively to negative experiences, thereby maintaining an overall positive motivational state. This profile is consistent with the adaptive profile hypothesized on the basis of traditional learning perspectives.

In addition, other profiles emerged, collectively accounting for a smaller yet meaningful proportion of the sample. Notably, two of these profiles were characterized by marked limitations in reflexive capacities, suggesting the presence of potentially vulnerable subgroups. Cluster 1 (4.9%) showed low levels of reflexivity (low RoE and very low reflexivity on practice), alongside high levels of negative affect and low levels of positive affect. This cluster, labeled *Inhibited–Unreflexive*, is thus characterized by a predominantly negative emotional state combined with a lack of reflexive capacities, representing the most critical pattern in the sample. This profile aligns with the hypothesized impaired profile.

However, the analysis identified an additional configuration (Cluster 2, 9.5%) with limited reflexive capabilities, which was not hypothesized. Individuals in this profile exhibit low levels of reflexivity (particularly regarding emotions) but show a distinct emotional pattern, with low negative affect and moderate positive affect. This pattern suggests a generally disengaged configuration and was therefore labeled the *Detached–Unreflexive* profile.

In contrast, the last profile (Cluster 3, 13.6%) displayed a distinctly constructive configuration. This profile, labeled *Generative–Reflexive*, was characterized by moderate levels of negative affect, high levels of positive affect, and elevated levels of both forms of reflexivity, representing the most resourceful pattern observed. Consistent with the hypothesized tension-enabled profile, this cluster suggests that combining the two reflexive forms not only mitigates the impact of negative experiences but also enhances positive ones.

### 4.3. Associations Between Emotional–Reflexive Profiles and Learning

To examine differences in transfer of learning across emotional–reflexive profiles, a one-way ANOVA was performed. Consistent with H2, each profile exhibited distinct associations with transfer of learning. Specifically, the *Generative–Reflexive* profile showed the highest mean learning transfer score (0.466), whereas the *Inhibited–Unreflexive* profile had the lowest (−0.888). The *Detached–Unreflexive* and *Balanced–Reflexive* profiles showed near-zero mean values (0.021 and −0.030, respectively). All reported scores are standardized values.

To further assess differences among profiles, a Kruskal–Wallis H test was conducted, followed by Dunn’s post hoc tests for multiple pairwise comparisons. The Bonferroni correction was applied to control the family-wise error rate (Calónico & Galiani, 2025). Table 5 summarizes the results of the post hoc comparisons. These results indicate statistically significant differences between most profile pairs. In particular, the *Generative–Reflexive* profile differed significantly from all other profiles (*p*.adj < 0.001), as did the *Inhibited–Unreflexive* profile when compared with both the *Detached–Unreflexive* and *Balanced Emotional–Reflexive* profiles (all *p*.adj < 0.001). By contrast, the comparison between the *Detached–Unreflexive* and *Balanced Emotional–Reflexive* profiles was not statistically significant (*p*.adj = 1.00), consistent with their similar mean scores.

Taken together, these findings highlight a clear gradient in learning outcomes associated with the combined configuration of emotional and reflexive dimensions. The *Generative–Reflexive* profile, characterized by the co-presence of positive affect and strong reflexive capacities, appears to be particularly conducive to transfer of learning, supporting the view that reflexivity amplifies the beneficial role of emotional resources. At the opposite end of the spectrum, the *Inhibited–Unreflexive* profile displayed the lowest transfer of learning scores, reflecting a pattern of cumulative disadvantage, in which high negative affect and limited reflexivity jointly constrain learning.

The interpretation of the intermediate profiles is more nuanced. The absence of significant differences between the *Detached–Unreflexive* and *Balanced–Reflexive* profiles suggests that moderate levels of reflexivity and balanced affect do not necessarily translate into enhanced learning outcomes. In this respect, the “normative” configuration (*Balanced*) does not emerge as optimally functional. The comparable performance of the *Detached–Unreflexive* profile, despite its low engagement in both emotional and reflexive processes, further indicates that disengagement does not inevitably impair learning, pointing to the possibility of alternative, less resource-intensive pathways to maintaining average levels of performance.

## 5. Discussion

The present study contributes to a more theoretically grounded understanding of how emotional and reflexive processes jointly shape the transfer of learning in workplace training contexts. By adopting a person-centered approach, the findings identify distinct configurations of these processes, suggesting that learning outcomes are not determined by isolated variables but linked to the patterned co-occurrence of these processes within individuals. This perspective aligns with contemporary views that conceptualize learning as an emergent, self-regulated process arising from the dynamic interplay between affective and cognitive-reflective systems (Rentzios et al., 2025).

From this standpoint, the four identified profiles can be interpreted as qualitatively different modes of engaging with learning experiences. In particular, the *Inhibited–Unreflexive* and *Generative–Reflexive* profiles delineate two theoretically meaningful poles. The former reflects a configuration in which elevated negative affect is neither balanced by positive affect nor supported by sufficient reflexive resources to process and regulate experience, thereby constraining the transformation of learning into practice. The latter, by contrast, exemplifies a generative configuration in which emotional and reflexive resources mutually reinforce each other: positive affect may sustain engagement, while reflexivity may facilitate the elaboration and integration of experience, ultimately facilitating transfer of learning.

The two intermediate profiles reveal more nuanced and less linear patterns, pointing to a complex relationship among emotional valence, reflexivity, and learning. The *Detached–Unreflexive* profile, characterized by medium-low emotional involvement (both positive and negative) and reduced reflexivity, showed comparatively low levels of transfer of learning, suggesting that low emotional engagement may constrain the processes through which experiences are transformed into meaningful learning (Rentzios et al., 2025; Lund Dean & Jolly, 2012). Interestingly, the *Balanced–Reflexive* profile, which was the most prevalent, showed moderate levels across all indicators and was not associated with higher transfer than the *Detached–Unreflexive* profile. This finding may reflect a form of “functional equilibrium” that, while stable, may lack the intensity or direction needed to foster deeper learning and transfer.

Taken together, these findings highlight that neither emotional detachment nor moderate levels of affect and reflexivity are associated with particularly favorable learning outcomes: while the *Detached–Unreflexive* profile displayed the lowest transfer of learning, the prevalence of the *Balanced–Reflexive* profile suggests that a substantial proportion of individuals may experience a moderate form of emotional–reflexive engagement that, although functional, seems to remain suboptimal, not paving the way to the highest levels of learning (Lund Dean & Jolly, 2012).

### 5.1. Theoretical Contribution

The present study makes three interrelated contributions to the literature. First, it provides an empirical identification of emotional–reflexive profiles through Latent Profile Analysis (LPA), extending the application of person-centered methods to workplace training research. Second, it offers preliminary evidence that negative affect is not uniformly associated with reduced transfer of learning and may coexist with positive learning outcomes in certain emotional–reflexive configurations, particularly when reflexive resources are relatively strong. Third, it introduces Reflexivity on Emotions (RoE) as a theoretically distinct construct that extends reflexivity beyond its traditional focus on practices and cognitions to encompass the emotional domain. Each of these contributions is elaborated below.

Adopting a person-centered, configurational perspective extends current understandings of workplace learning by foregrounding the joint and interdependent roles of emotions and reflexivity. Rather than conceptualizing learning as the outcome of isolated factors, this approach emphasizes that individuals’ learning trajectories are associated with patterns of co-occurring experiences and capabilities. In this sense, learning is not merely a function of “more” or “less” of a given variable, but of how different dimensions—such as positive and negative emotions, reflexivity on practice, and reflexivity on emotions—are combined within individuals.

Person-centered approaches are increasingly recognized as valuable for capturing heterogeneity in learning processes. Building on this perspective, the present study advances the literature by extending its application to workplace learning and by integrating reflexivity and emotional experiences within a unified analytical framework. Whereas prior research has primarily examined metacognitive skills without explicitly incorporating reflexivity (Altunay & Özdemir, 2025) or has embedded reflexivity within broader models of self-regulated learning while giving limited attention to the emotional dimension (Schel & Drechsel, 2025), this study brings these strands together. By jointly considering reflexivity on practices, reflexivity on emotions, and both positive and negative affect, this approach challenges reductionist perspectives that treat cognition, metacognition, and affect as separable domains. Instead, it supports a view of learning as an emergent property of dynamic configurations, where cognitive and emotional processes are closely intertwined and shape whether and how learning is effectively translated into practice.

A second contribution concerns the role of negative emotions in workplace learning. Existing research has generally emphasized the facilitating role of positive emotions in learning processes, while negative emotions have often been viewed as obstacles to learning and performance. The present findings suggest a more nuanced picture. Specifically, the Generative–Reflexive profile, characterized by elevated negative affect alongside strong reflexive resources, was associated with the highest levels of transfer of learning. Although the cross-sectional nature of the study precludes conclusions regarding underlying mechanisms, this pattern is consistent with the idea that negative emotions are not necessarily detrimental to learning outcomes. Rather, when accompanied by adequate reflexive resources, they may coexist with positive learning outcomes and potentially serve as signals that stimulate deeper engagement with work experiences, playing a potentially constructive role in learning and development.

Finally, this study extends organizational learning theory by broadening the target of reflexivity beyond the well-established link between reflexivity and learning (Gherardi et al., 2018; Beers & van Mierlo, 2017), as well as potential dysfunctional cycles (Heikkilä et al., 2012). The findings suggest that profiles characterized by lower levels of RoE tend to be associated with lower levels of transfer of learning, underscoring the relevance of reflexive capabilities across both domains. In this sense, RoE may represent a distinct capability through which individuals become aware, interpret, and respond to emotional experiences encountered in the workplace, transforming potentially disruptive emotions into meaningful inputs for learning. More broadly, the findings are consistent with the view that emotional experiences may constitute an important object of reflexive inquiry and that learning processes may benefit not only from reflection on actions and practices, but also from reflection on the emotions that accompany them. By extending reflexivity into the emotional domain, the present study contributes to a more granular understanding of how individuals learn from workplace experiences.

### 5.2. Practical Implications

Acknowledging RoE as a core capability for navigating emotional experiences in training settings holds significant practical implications for organizations. First, organizations should promote a cultural perspective that frames emotions not merely as sources of strain or disruptions, but as valuable resources for sensemaking, adaptation and learning, that inform professional practice. Embedding this perspective into organizational values and policies can legitimize emotional awareness and reflexive practice as essential components of professional competence.

Second, training programs should integrate RoE-focused interventions to strengthen employees’ ability to recognize, sense-make, and learn from emotional experiences. This is particularly relevant in emotionally charged professions, characterized by frequent exposure to highly involving or conflictual interpersonal interactions—such as healthcare, customer service, education, and correctional policing (Cova & Farnese, 2025)—where unmanaged emotions may contribute to burnout and impaired performance. For instance, *formal tutors* can play a key role in creating reflective, psychologically safe learning environments (Moore & Wang, 2017; Tsuei et al., 2019), helping trainees disentangle emotionally challenging experiences and transform them into learning opportunities rather than suppressing them in response to occupational or institutional pressures. Complementary strategies—such as structured debriefing sessions following critical incidents, reflective coaching, peer reflection groups, supervisory reflection sessions, and reflective journaling—may support employees in exploring the emotional meaning of challenging work experiences, deriving meaning from them, and translating these insights into adaptive professional practices. Together, these practices can foster learning environments in which emotions are recognized not merely as potential confounding factors but as valuable sources of insight, growth, and professional development, making RoE a fundamental capability of professional learning.

More broadly, the identification of distinct emotional–reflexive profiles suggests that organizations may benefit from moving beyond one-size-fits-all approaches toward a more differentiated, targeted design. A person-centered approach enables organizations to tailor development initiatives to individuals’ specific configurations of emotional and reflexive capabilities, thereby enhancing training effectiveness. In this regard, leaders and supervisors play a particularly important role. By developing their own RoE capabilities, they may be better equipped to establish psychologically safe environments conducive to open dialogue, collective learning, and innovation.

Overall, these findings highlight the importance of interventions that not only mitigate the potential costs of negative emotions but also actively cultivate critical inquiry capacities and constructive emotional engagement. Such interventions may contribute to individual development, workplace well-being, and organizational learning.

### 5.3. Limitations and Further Research

Despite these contributions, some limitations should be acknowledged. First, the cross-sectional nature of the study precludes causal conclusions regarding the relationships among emotions, reflexivity, and transfer of learning. The observed associations should therefore be interpreted as correlational rather than causal. Future research would benefit from longitudinal and prospective designs capable of examining how emotional–reflexive configurations evolve over time and how they relate to subsequent learning outcomes.

Second, the study relies exclusively on self-reported data, which may introduce common-method bias and social-desirability effects. This concern is particularly relevant to measuring transfer of learning, as participants in training contexts may be inclined to overestimate the extent to which they have acquired and can apply new knowledge and skills, potentially inflating the observed associations. Future research should complement self-reported measures with behavioral observations, supervisor ratings, or objective performance indicators, in order to strengthen the construct validity of the observed relationships.

Third, the measurement of positive affect represents a further limitation. The short form of the PANAS used in this study exhibited relatively low reliability for positive affect, raising concerns about its accuracy. This limitation may have attenuated the observed associations involving positive emotions, potentially leading to conservative estimates of their role in shaping the emotional–reflexive configurations. Future studies should adopt a broader range of positive emotions to better capture the complexity of affective experiences involved in learning processes.

Fourth, although the RoE scale demonstrated acceptable psychometric properties in the present study, additional validation work is needed. Future research should further examine its convergent and discriminant validity, test measurement invariance across different occupational and cultural contexts, and provide additional evidence regarding its stability and generalizability.

A further limitation concerns the analytical strategy used to examine differences in transfer of learning across latent profiles. Although profile comparisons were conducted using participants’ most likely class membership, more advanced three-step procedures, such as the BCH approach, are generally recommended because they explicitly account for classification uncertainty when estimating relations between latent profiles and distal outcomes. While the high entropy of the retained solution suggests satisfactory classification accuracy, future studies should replicate these findings using BCH or similar model-based approaches to provide more robust estimates of profile differences.

Finally, the sample was drawn from a highly specific occupational context (namely, Italian correctional officers), potentially limiting the generalizability of the findings across cultural and occupational settings. Prisons represent a particularly valuable context for examining emotional–reflexive processes because correctional work is characterized by high emotional demands and frequent exposure to emotionally challenging situations. At the same time, however, the highly regulated normative environment may discourage overt emotional expression, fostering emotional containment as a professional norm.

Such contextual characteristics may have influenced the emotional–reflexive configurations identified in the present study. Relatedly, although the LPA solution showed satisfactory fit indices and classification accuracy, the predominance of the *Balanced–Reflexive* profile suggests that the observed pattern may partly reflect the relative homogeneity of this organizational context. Specifically, the procedural constraints and normative expectations associated with correctional work may have encouraged a relatively uniform emotional–reflexive orientation among trainees, contributing to the concentration of participants within a single profile. Consequently, the identified profiles should be interpreted as contextually situated configurations rather than as universally generalizable psychological typologies. Future studies should examine whether the *Balanced–Reflexive* profile represents a normative baseline or a context-specific adaptation to the demands of correctional work. Replication with larger, more heterogeneous samples, as well as across occupational settings with varying levels of emotional demands, is therefore needed to assess the stability, robustness, and generalizability of the identified configurations. Longitudinal research would also help clarify the developmental trajectories and stability of these profiles over time.

## 6. Conclusions

Reflexivity on emotion (RoE) emerges as a unique metacognitive capability that plays a crucial role not only in managing affective states but also in shaping how learning is transferred. The person-centered approach adopted in this study further reveals distinct emotional–reflexive profiles, underscoring the intertwined role of emotions and reflexivity in workplace learning, and therefore the importance of fostering both reflexive awareness and meaningful emotional engagement to support effective learning and its transfer into the workplace.

## Figures and Tables

**Table 1:** Second-order confirmatory factor analysis loadings for reflexivity constructs.

Latent Variables	Estimate	Std. Err	z-Value	*p* (>|z|)	Std. Lv.	Std. All.
Reflexivity (total)						
Reflexivity on Emotions	1.000				2.840	2.840
Reflexivity on Practice	0.082	0.010	8.255	0.000	0.223	0.223

**Table 2:** Descriptions and intercorrelations among the variables of the study.

Variable	Mean	SD	SE	1	2	3	4	5
1. Reflexivity on Practice	3.95	0.510	0.020	—				
2. Reflexivity on Emotions	3.89	0.624	0.025	0.580 ***	—			
3. Negative Affect	2.19	0.782	0.031	−0.030	0.091 *	—		
4. Positive Affect	4.19	0.607	0.024	0.210 ***	0.212 ***	−0.247 ***	—	
5. Transfer of Learning	4.16	0.656	0.026	0.306 ***	0.238 ***	−0.186 ***	0.304 ***	—

*Note.* * *p* < 0.05, *** *p* < 0.001.

**Table 3:** Fit indices for the seven estimated solutions of emotional–reflexive profiles.

Number of Profiles	AIC	BIC	SABIC	Entropy
1	6925	6960	6934	1.000
2	6685	6742	6701	0.717
3	6559	6639	6581	0.758
4	6328	6429	6356	0.896
5	6214	6337	6249	0.822
6	6144	6289	6185	0.845
7	6055	6223	6102	0.856

*Note*: AIC = Akaike’s information criterion; BIC = Bayesian information criterion; SABIC = Sample-size Adjusted Bayesian Information Criterion.

**Table 4:** Characteristics of the emotional–reflexive profiles based on standardized indicator scores.

Profile	Reflexivity on Practice	Reflexivity on Emotion	Negative Affective	Positive Affective
Inhibited–Unreflexive	−2.237	−1.139	0.094	−1.796
Detached–Unreflexive	−0.732	−1.832	−0.460	−0.080
Generative–Reflexive	1.490	1.430	−0.002	0.604
Balanced–Reflexive	−0.034	0.056	−0.003	0.021

**Table 5:** Pairwise comparisons of transfer training scores using Dunn’s test with Bonferroni correction.

Comparison	Z	*p*.unadj	*p*.adj
1-2	−5.32	<0.001	<0.001
1-3	−11.26	<0.001	<0.001
2-3	−7.02	<0.001	<0.001
1-4	−7.04	<0.001	<0.001
2-4	−0.94	0.347	1.000
3-4	8.95	<0.001	<0.001

*Note*: *p*.adj = Bonferroni-corrected *p*-value.

## Data Availability

Data are available from the authors upon reasonable request.
